# Clinical Analysis and Management of Gestational Trophoblastic Diseases: A 90 Cases Study

**Published:** 2009-12

**Authors:** Ben Temime Riadh, Chechia Abdellatif, Hannachi Wissal, Attia Leila, Makhlouf Taher, Koubaa Abdelhamid

**Affiliations:** *Department of obstetrics and gynecology, Charles Nicolle University Hospital Tunis, Tunisia*

**Keywords:** hydatiform mole, human chorionic gonadotropin, ultrasonography, gestational trophoblastic tumors, chemotherapy

## Abstract

**Objective:**

The aim of the study was to identify the incidence, diagnosis, therapeutic and histological particularities of molar pregnancies and to evaluate our management of gestational trophoblastic tumors (GTT) according to the recommendations of FIGO.

**Methods:**

This was a retrospective study of 90 patients who were diagnosed with molar pregnancy from January 1991 to December 2007. After remission, post molar pregnancy surveillance was continued for one year. Patients whose condition required chemotherapy for GTT were attributed a FIGO/WHO score.

**Results:**

Molar pregnancy occurred in 90 women. The frequency of molar pregnancy was 1 per 1124 pregnancies. The mean age was 32.21 years. Molar pregnancies were more frequent in pauciparous patients (52.24%). At diagnosis, the median gestational age was 13 weeks. The main presenting symptom was metrorrhagia (90%). Treatment consisted in uterine evacuation by suction curettage. Histological findings were complete mole in 66.66% of the cases and partial mole in 33.33% of the cases. 81 patients (90%) achieved remission without chemotherapy and 9 patients (10%) had FIGO stage I GTT. They achieved remission with a monochemotherapy.

**Conclusion:**

The practice of ultrasonography in the first trimester of pregnancy allows an early diagnosis of molar pregnancy and an adequate treatment and follow-up.

## INTRODUCTION

Molar pregnancies account for most malignancies that originate from the placenta and are characterized by significant hydropic enlargement and variable trophoblastic hyperplasia. They are classified into partial and complete subtypes according to histopathologic and genetic criteria. Suction curettage is the preferred method of evacuation. Strict compliance with post molar pregnancy surveillance is essential because of the potential development of gestational trophoblastic tumors (GTT). Important advances have been made in the diagnosis, treatment and follow-up of patients with molar pregnancy and GTT.

## MATERIALS AND METHODS

This was a retrospective study of 90 patients who were diagnosed with molar pregnancy during a 17-year period from January 1991 to December 2007. Clinical, ultasonographic, Human chorionic gonadotrophin (HCG) levels, therapeutic, and histological data were analyzed. After suction evacuation of the molar pregnancy, patients were monitored in a standard fashion, including serial serum and urinary HCG assays. After remission, post molar pregnancy follow up was continued for one year. Nine patients presented a gestational trophoblastic tumor. FIGO/WHO revised classification was used. Patients who presented a GTT had a low-risk non metastatic GTT in all cases. Treatment consisted in a single agent chemotherapy based either on D actinomycin (1.25 mg/m^2^ intravenous repeated every 2 weeks) or Methotrexate (0.4 mg/Kg intramuscular for 5 days repeated every 2 weeks). Patients whose condition required chemotherapy for GTT had two years of follow up examination.

## RESULTS

Molar pregnancy occurred in 90 women. The frequency of molar pregnancy was 1 per 1124 pregnancies. The median age was 31.21 years (range 17–51 years). 30% of our patients were aged over 35 years. 74 Patients (82.22%) originated from the north of Tunisia, 12 patients (13.33%) from the centre and 4 from the south (4.44%). The mean parity was 2.05 (range 0–10). Molar pregnancies were more frequent in nulliparous (28.88%) and pauciparous (52.24%) patients. The pregnancy that preceded directly the molar pregnancy was a normal term pregnancy in 50 cases, a miscarriage in 12 cases, a voluntary abortion in 3 cases and a molar pregnancy in 1 case. A history of toxaemia and hypertension was reported in 6 cases. A history of diabetes mellitus was reported in 4 cases. A history of molar pregnancy was reported in 3 cases (3.33%): ten, four and two years before. 39 patients had used contraceptive methods before the molar preganacy: an intrauterine device in 26 cases (28.88%) and an estro-progestative pill in 13 cases (14.44%). The blood group was “0” in 57.77%, “A” in 20%, “B” in 14.46% and “AB” in 7.77% of the cases.

At diagnosis, the median gestational age was 13 weeks (range: 5–33 weeks). 82.24% of the patients were diagnosed between 8 and 16 weeks of pregnancy. Patients presented with metrorrhagia in 90% of the cases, abdominal distension in 48.88% of the cases and hyperemesis gravidarum in 27.77% of the cases. Four patients presented with a first trimester miscarriage. One patient reported an absence of fetal active movements and two patients were asymptomatic; the diagnosis was made at first trimester ultrasonography.

The physical examination showed clinical sings of anaemia in 20 patients (22.22%). The uterine size was palpably larger than the expected gestational age in 49 patients (54.44%). Theca lutein cysts were clinically palpable and bilateral in 5 patients (5.55%). No Pre-eclampsia and no hyperthyroidism were observed.

Ultrasounds were performed before pregnancy evacuation in 86 patients (95.55%). The other 4 patients presented with a miscarriage. Ultrasounds showed typical diffuse echogenic vesicular pattern, evoking a complete hydatiform mole in 68 patients (79.06%). They showed an excessively enlarged placenta with cystic spaces and a gestational sac which was either empty or contained amorphous echos, evoking a partial hydatiform mole in 6 patients (6.97%). Finally, ultrasounds could not make the difference between a molar pregnancy and a missed abortion in 12 cases (13.97%). Moreover, ultrasounds showed bilateral luteal cysts in 8 cases (9.3%) and unilateral cysts in 5 cases (5.81%) with a 5cm average size.

Markedly elevated HCG levels are commonly seen in patients with molar pregnancy. A pre evacuation urinary HCG measurement was performed in 60 patients (before 2000). 89.99% of these patients had a urinary HCG level greater than 50000 IU/l and 23.33% of them had a level greater than 400000 IU/l. Serum β HCG measurement was performed in 26 patients. The level was greater than 50000 IU/l in 61.52% of them. In patients who presented with a miscarriage, a urinary HCG measurement was performed the following day and the levels ranged from 200000 IU/l to 400000 IU/l.

Suction curettage was performed in all cases. It was performed under sonographic control in 74 cases. An oxytocin infusion was started before anesthesia induction in order to increase myometrial tone and decrease blood loss. A cervical dilation with Hegar bougies was necessary in 52 cases. A cervical maturation using prostaglandins was necessary in 3 patients who were suspected to have a second trimester missed abortion. All patients had pre evacuation antibiotic prophylaxis using either Amoxicillin or Doxycycline. Nine patients who were Rh negative received Rh immune globulin at the time of evacuation. No prophylactic chemotherapy was used.

Histological findings were complete hydatiform mole in 60 cases (66.66%) and partial hydatiform mole in 30 cases (33.33%).

After evacuation, the follow up was clinical, biological and by ultrasounds. Patients were monitored with weekly hCG levels until non-detectable for three weeks and then monthly hCG levels until non-detectable for one year. A chest radiograph was performed in all patients. It was normal in all cases. All patients were encouraged to use an effective oral contraceptive: an estro-progestative pill in 74 cases (82.22%) and a macroprogestin in 16 cases (17.77%). The evolution was favourable in 81 patients (90% of the cases) characterized by the disappearance of vaginal bleeding averagely within 6 days and a uterine involution within 15 days. HCG levels became undetectable within 6 weeks (range: 4 to 10 weeks).

Nine patients (10%) developed a non metastatic gestational trophoblastic tumor. The follow up showed an HCG level plateau recorded over a 3-week duration in 5 cases and an HCG level increase of 3 values over a 2-week duration in 4 cases. Transvaginal ultrasonography showed hypoechoic intra uterine areas with rich colour flow Doppler vascularity in 4 cases and mixed echogenicity myometrial nodules evoking a myometrial invasion in 3 cases. Clinical examination showed no vaginal metastases. The chest radiographs and the liver ultrasounds were normal in all cases. The gestational trophoblastic tumor occurred after a complete hydatiform mole in 8 cases and a partial mole in 1 case. FIGO/WHO revised classification was used. Patients who presented a GTT had a FIGO/WHO score between I:2 and I:4 in all cases. Treatment consisted in a single agent chemotherapy based on D actinomycin (1.25mg/m^2^ intravenous repeated every 2 weeks) in 6 cases and Methotrexate (0.4mg/Kg intramuscular for 5 days repeated every 2 weeks) in 3 cases. Treatment tolerance was acceptable. βHCG serum level became undetectable with 6 cycles averagely. A two-year follow-up showed a complete remission with no relapse in all cases.

## DISCUSSION

The frequency of molar pregnancy varies largely from a country to another. In Asian countries this frequency is seven to ten times greater than that reported in North America or in Europe (1, 2). In Taiwan for instance, hydatiform mole occurs in 1 per 125 pregnancies whereas in the United States it occurs in 1 per 1500 pregnancies (1, 2). In our Tunisian series, the frequency of molar pregnancy is low, accounting for 1 per 1124 pregnancies. Many risk factors, including maternal age, poor nutrition, as well as the effects of prior pregnancies, consanguinity, ABO blood group, contraceptive methods and environmental factors have all been reported in literature (1, 3, 4). In our series, the maternal age has consistently been identified as an important risk factor: women older than age 40 years have substantially higher incidence rates. Moreover a history of a prior spontaneous abortion and especially a history of a previous hydatiform mole appear to be strong and well established risk factors predisposing to another molar pregnancy. However, Blood group A, classically reported in literature was only present in 20% of our cases.

The clinical presentation of hydatiform mole has significantly changed in the last 20 years and this effect was attributed to the early diagnosis (3, 5). The widespread use of ultrasounds in pregnancy contributed to this change. Vaginal bleeding is a common event, being present in about 70 to 97% of the patients in most series (5–8). An early diagnosis of hydatiform mole led to the uncommon presentation of severe pre-eclampsia, pulmonary embolism, severe anemia and large lutein cysts. The results of our study are consistent with those obtained from a literature review (Table 1).

**Table 1:** Clinical features of hydatidiform mole at presentation

Author	Period	N	Vaginal bleeding	Increased uterine size	Hyperemesis gravidarum	Theca lutein cysts	Pre-eclampsia

Soto-Wright (3)	1965–1975	306	97%	51%	26%	-	27%
Soto-Wright (3)	1988–1993	74	84%	28%	8%	9%	1%
Berkowitz (1)	1979–1984	81	73%	4%	N/A	0	3%
Coukos (7)	1989–1997	24	75%	54%	0	0	0
Gemer (8)	1988–1998	41	58%	15%	2%	-	0
Mangili (5)	1970–1982	311	74%	51%	34%	21%	3%
Mangili (5)	1992–2004	189	51%	29%	26%	13%	1%
Our series	1991–2007	90	90%	54.44%	27.77%	5.55%	0

Our understanding of the cytogenetics, epidemiology, pathology and clinical management of hydatidiform mole has advanced considerably these last years. It is now well recognized that molar pregnancy comprises two distinct entities, complete and partial, which differ on the basis of chromosomal pattern, gross and microscopic histopathology and clinical presentation (1, 2, 9).

Both Ultrasonography and the dosage of β HCG serum levels are sensitive and reliable tools for detecting molar pregnancy. The sonographic characteristic findings are the snowstorm appearance of uterine contents in case of a complete mole and focal cystic changes in the placenta (1, 10). However, it is reported that ultrasonography can detect a molar pregnancy before evacuation in less than 60% of cases (5). Thus, the histologic examination of the products of conception from first-trimester miscarriage remains the gold standard for diagnosis of HM. It is reported that 70% of partial mole and that 16% of complete mole were unsuspected if routine histologic examination was not performed after evacuation (5).

Some recommendations about the management of molar pregnancies were reported by the FIGO (2, 9, 11). The hydatidiform mole is surgically evacuated as soon as possible after diagnosis. If haematologic, thyroid or pulmonary problems are present, these are treated. Evacuation should be done by suction curettage with accompanying syntocinon infusion. The cervix may be dilated gently and slowly. With complete mole, a 9 mm or 10 mm suction curettage usually suffices and greater dilatation of the cervix is usually not necessary (1, 2, 9). A careful, “light” sharp curettage should be performed following the suction procedure to ensure the uterus has been completely evacuated. Hysterectomy may be done in patients who have finished childbearing (1, 2, 9). All these recommendations were carefully followed in our series. Serial serum hCG monitoring is important immediately after evacuation (1, 2, 6, 9). A weekly monitor till level returns to normal would enable early diagnosis of gestational trophoblastic neoplasia (GTN). Subsequent monitoring after hCG has returned to normal could be spaced to once every 2 weeks then 4 weeks for 6 to 12 months. A reliable contraceptive method is preferred. The use of oral contraceptives is safe (1, 2, 11).

In 2000, criteria for the diagnosis of GTN following molar pregnancy have been agreed and were published in the FIGO Gynecology Oncology Report (1, 2, 9, 11). GTN may be diagnosed when the plateau of human chorionic gonadotrophin lasts for four measurements over a period of 3 weeks or longer; when there is a rise of hCG of three weekly consecutive measurements or longer; when there is histologic diagnosis of choriocarcinoma and finally, GTN is diagnosed when the hCG level remains elevated for 6 months or more. An assay for HCG sensitive to 2mIU/ml or less is essential for the follow-up (1, 2).

Ten to 20% of patients presenting a complete hydatiform mole and 3.5 to 5% of patients presenting partial moles will develop a GTN (1, 2, 12–15). Our results are consistent with those of literature (Table 2). A revised staging of GTN was adopted by the FIGO and published in 2002. It recommended allocation of GTN into low and high risk groups only using the cutoff point of six inclusive. (1, 2, 16). This revised staging was used in all our patients (after 2002). They all had FIGO/WHO score between I:2 and I:4.

**Table 2:** Frequency of post molar gestational trophoblastic tumors

Author	N	Non Metastatic GTT (%)	Metastatic GTT (%)	Total GTT (%)

Berkowitz (1)	858	14.7	4	18.7
Lurain (12)	738	16.3	3	19.3
Curry (13)	347	16.6	3.5	20.1
Morrow (14)	121	23.1	3.3	26.4
Kohorn (15)	127	26	3.1	29.1
Our series	90	10	0	10

Finally, concerning the treatment of GTN: Methotrexate with or without folinic acid rescue remains the most effective single agent in the treatment of low risk GTN. Actinomycin D is an effective alternative (11, 12, 15). Both molecules were used in our series with a good response in all cases. The treatment of high risk GTN is based on multiple chemotherapy: EMA-CO (etoposide, methotrexate, actinomycin, cyclophosphamide and vincristine) has been used widely with 86% 5 years survival (1, 12, 16–18). EMA-EP (etoposide, methotrexate, actinomycin plus etoposide and cisplatin) is an effective alternative in case of resistance. (1, 12, 16–18).

## CONCLUSION

The clinical presentation of hydatiform mole changed over the last years because of the widespread of first trimester ultrasonography. As reported in our series, molar pregnancy is diagnosed earlier (median gestational age was 13 weeks at diagnosis). This led to the uncommon presentation of severe pre-eclampsia, pulmonary embolism and severe anaemia. Important advances continue to occur in both our understanding and management of gestational trophoblastic diseases. The FIGO recommendations allowed the unification of diagnostic criteria and prognostic factors. The importance of our study is to stress on the curability of GTT if diagnosed on time. Though there is not a high risk group of patients in our series, the review of literature shows a five-year survival over 80%. Finally, the creation of a reference centre will improve and standardize the management of GTN and will assure an appropriate treatment according to the risk groups, resulting in the best outcome.
